# Three‐dimensional deep neural network for automatic delineation of cervical cancer in planning computed tomography images

**DOI:** 10.1002/acm2.13566

**Published:** 2022-02-22

**Authors:** Yi Ding, Zhiran Chen, Ziqi Wang, Xiaohong Wang, Desheng Hu, Pingping Ma, Chi Ma, Wei Wei, Xiangbin Li, Xudong Xue, Xiao Wang

**Affiliations:** ^1^ Department of Radiation Oncology Hubei Cancer Hospital TongJi Medical College Huazhong University of Science and Technology Wuhan Hubei China; ^2^ Key Laboratory of Artificial Micro and Nano‐structures of Ministry of Education Center for Theoretical Physics School of Physics and Technology Wuhan University Wuhan China; ^3^ Department of Radiation Oncology Rutgers‐Cancer Institute of New Jersey Rutgers‐Robert Wood Johnson Medical School New Brunswick New Jersey USA

**Keywords:** automatic delineation, cervical cancer, CTV, deep learning model

## Abstract

**Purpose:**

Radiation therapy is an essential treatment modality for cervical cancer, while accurate and efficient segmentation methods are needed to improve the workflow. In this study, a three‐dimensional V‐net model is proposed to automatically segment clinical target volume (CTV) and organs at risk (OARs), and to provide prospective guidance for low lose area.

**Material and methods:**

A total of 130 CT datasets were included. Ninety cases were randomly selected as the training data, with 10 cases used as the validation data, and the remaining 30 cases as testing data. The V‐net model was implemented with Tensorflow package to segment the CTV and OARs, as well as regions of 5 Gy, 10 Gy, 15 Gy, and 20 Gy isodose lines covered. The auto‐segmentation by V‐net was compared to auto‐segmentation by U‐net. Four representative parameters were calculated to evaluate the accuracy of the delineation, including Dice similarity coefficients (DSC), Jaccard index (JI), average surface distance (ASD), and Hausdorff distance (HD).

**Results:**

The V‐net and U‐net achieved the average DSC value for CTV of 0.85 and 0.83, average JI values of 0.77 and 0.75, average ASD values of 2.58 and 2.26, average HD of 11.2 and 10.08, respectively. As for the OARs, the performance of the V‐net model in the colon was significantly better than the U‐net model (*p *= 0.046), and the performance in the kidney, bladder, femoral head, and pelvic bones were comparable to the U‐net model. For prediction of low‐dose areas, the average DSC of the patients’ 5 Gy dose area in the test set were 0.88 and 0.83, for V‐net and U‐net, respectively.

**Conclusions:**

It is feasible to use the V‐Net model to automatically segment cervical cancer CTV and OARs to achieve a more efficient radiotherapy workflow. In the delineation of most target areas and OARs, the performance of V‐net is better than U‐net. It also offers advantages with its feature of predicting the low‐dose area prospectively before radiation therapy (RT).

## 1 INTRODUCTION

The most common type of cervical cancer is squamous cell carcinoma, followed by adenocarcinoma and adenosquamous carcinoma. Small cell carcinoma and clear cell carcinoma are rare types of cervical cancer.^1^ Due to the sensitivity to radiation of squamous cell carcinoma,^2^ most cervical cancers can be treated with external beam radiotherapy and brachytherapy to achieve the tumor‐fatal maximum radiation dose to the cervix.^3^ Ng et al.^4^ indicated that insufficient dose of modern intensity‐modulated radiation therapy (IMRT) is still one of the important factors affecting the therapeutic effect, and that one of the key factors of limiting the dose of radiotherapy is the delineation of the tumor target area and related organs at risk (OARs). However, the accuracy of manual delineation mostly depends on the clinical experience of physicians, as different physicians may have different contouring standards.^5^ In addition, manual delineation is a very labor‐intensive and time‐consuming process, which greatly increases the workload of physicians. An accurate and efficient method to automatically segment target areas and OAR is in great need to speed up the process of contouring and reduce the time needed for treatment planning.

Atlas‐based automatic segmentation^6^, ^7^ is a common method in the routine clinical practice. This method uses deformation registration to match a new image with a selected set of contours in the database.^8^ However, for images in the pelvic region, due to the differences in stages and treatment schemes of different patients,^9^ discrepancy in size, shape, texture grayscale, and relative position can be observed, which limits the performance of this method.

Inspired by the successful application of deep learning in computer vision, the deep learning methods have been introduced to radiotherapy. Deep learning represented by convolutional neural networks (CNN) has made rapid progress in the fields of computer science and medicine.^10^, ^11^, ^12^, ^13^, ^14^ Automatic segmentation by deep learning has shown performance comparable to or even beyond manual delineation in computed tomography (CT),^15^ magnetic resonance imaging (MRI),^16^ and ultrasound^17^ images at several anatomic sites, such as brain, liver, rectum, bladder, and anterior glands. However, for cervical cancer patients, due to the high overlap of pelvic organs and the ambiguity of the interface between tumor and normal tissue,^18^ it is still a challenge to automatically segment pelvic targets through deep learning.

This study aims to examine the automatic segmentation of pelvic clinical target volume (CTV) and OARs by using three‐dimensional (3D) deep learning model and compare the results to the 2D model. In addition, for young patients with cervical cancer, in order to preserve the patient's ovarian function, the surgeons will transpose the ovary and its arteries and veins to the abdomen during hysterectomy. With this in mind, during postoperative radiotherapy, the radiation dose at the new ovarian position needs to be controlled within a safe range. So, we creatively propose to use certain dose distribution of the radiotherapy plan as the goal of deep learning to explore the feasibility of predicting the position of ovarian transposition before surgery.

## 2 MATERIAL AND METHODS

### 2.1 V‐net model structures

Most methods of CNNs can only process 2D images, while most of the medical data used in clinical practice are 3D.^19^ Under this situation, V‐net provides a 3D image segmentation method.

**FIGURE 1:**
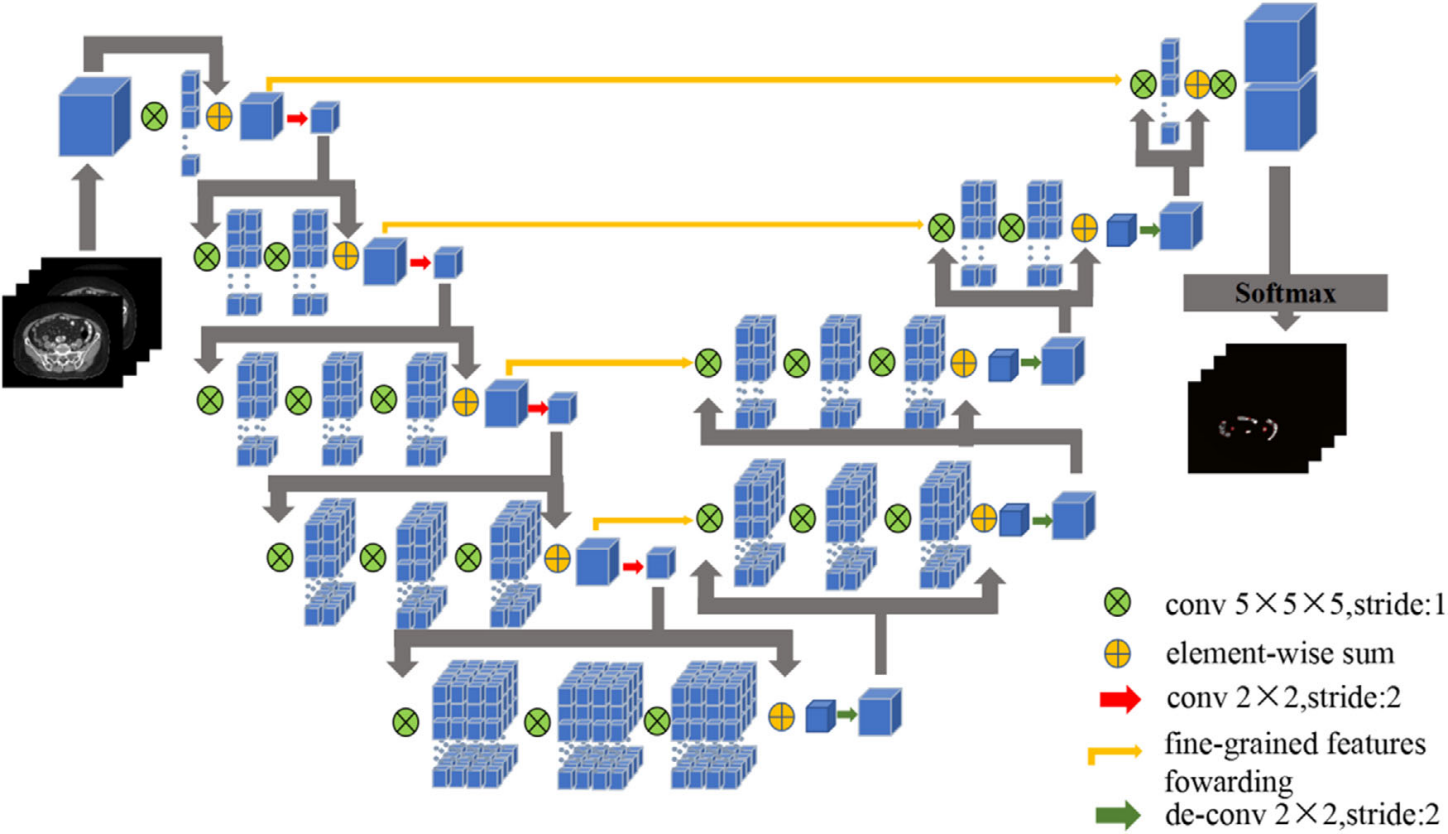
The structure of V‐net

As shown in Figure 1, the left part of the network consisted of contracting paths, while the right part expanded the signal until it reached its original size. All convolutions used proper padding. The network on the left was divided into different stages by operations at different resolutions. Each stage consisted of 1–3 convolutional layers. The convolution process of each stage captured the residual features like ResNet.^20^ The input feature map was added to the original input point by point after several convolutions and nonlinear activations, and then down‐sampled. The size of the convolution kernel for each convolution was 5×5×5, and the down‐sampling process was using a 2×2×2 convolution kernel with a step size of 2. Since the latter operation only extracted features in non‐overlapping 2×2×2 convolution blocks, the size of the resulting feature map was halved. The network on the right part extracted features and expanded the spatial support of the low‐resolution feature map in order to collect and combine the necessary information to achieve the volume segmentation in two channels which is a binary classification. The two feature maps, with kernel size of 1×1×1 and output being the same size as the input, calculated by the last convolutional layer were converted into the probability segmentation of the foreground and background regions by applying the softmax function.

In the processing of medical images, the size of region of interest (ROI) is usually relatively small compared to the whole image, which will cause the network learning process to fall into the local minimum of the loss function, resulting in a network whose prediction is heavily biased toward the background, whereas missing or partially detect foreground elements. Traditional deep learning methods use a loss function based on sample weighting, which makes the foreground area more important than the background area in learning. V‐net proposed a new objective function based on Dice‐coefficient. Using this formula, we do not need to assign weights to samples of different categories, and we can establish the correct balance between foreground voxels and background voxels. The Dice‐coefficient *D* between two binary volumes is expressed as follows:

(1) $$D=\frac{2\sum {}_{i}^{N}p_{i}g_{i}}{\sum {}_{i}^{N}p_{i}^{2}+\sum {}_{i}^{N}g_{i}^{2}},$$

where the sums run over the *N* voxels of the predicted binary segmentation volume *p_i_* ∈ *P* and the ground truth binary volume *g_i_* ∈ *G*.

### 2.2 Training data

A total of 130 cervical cancer patients who received radiotherapy in the Department of Radiotherapy of Hubei Cancer Hospital from 2017 to 2020 were included in this study. All patients underwent CT scan (Philips, Big bore brilliance) with contrast in the supine position. CT images were obtained with 3 mm slice thickness, with size of 512 × 512 pixels and resolution of 0.98 mm × 0.98 mm. The target area CTV and OARs of all patients were delineated by the chief physician of the same department on varian eclipse treatment planning system (TPS), based on female pelvic normal tissue contouring atlas for Radiation Therapy Oncology Group.^21^ In addition, the different dose areas in the radiotherapy plan were also outlined as new ROIs, by directly converting isodose lines in clinical plans to structures in the TPS. In this study, all treatment plans were planned with volumetric modulated arc therapy (VMAT) technology using 6 MV photon beams, and anisotropic analytical algorithm (AAA) algorithm was used for the dose calculation.

In the training process, 90 cases were randomly selected from 130 patients as the training set to adjust the training parameters of the automatic segmentation model, and the remaining 40 cases were divided into the verification set and the testing set to evaluate the performance of the model. Prior to the training process, the CT images were normalized and resampled to ensure the standardization of the dataset, which unified the values while maintaining the original distribution, and standardized the voxel spacing. The CNN algorithm was implemented based on the Version 2 of Tensorflow, and also used for model training, evaluation, and error analysis. The NVIDIA GeForce RTX 2070 with 8 GB capacity was utilized to facilitate the process. A batch size of 16 was used for the U‐net model and a batch size of 8 was used for the V‐net model. The proposed V‐net model was trained using Adam optimizer for 100 epochs, with the initial learning rate of 0.0003. The training time needed for each OAR was around 3 and 4 h for U‐net and V‐net, respectively. After training, it took about 1 s for both networks to delineate one OAR for new input CT images.

### 2.3 Evaluation indicators

Dice similarity coefficient (DSC), Jaccard index (JI), Average surface distance (ASD) and Hausdorff distance (HD) were used to evaluate the accuracy of segmentation in the testing dataset.

DSC is defined as the ratio of the overlap between the predicted contour A and the real contour B. The formula is as follows:

(2) $$\mathrm{DSC}\left(A,B\right)=\frac{2\left|A\cap B\right|}{\left|A\right|+\left|B\right|}.$$

The value is between 0 and 1. The larger the value, the higher the degree of overlap between the segmented image and the manual outline.

JI is the ratio of the intersection elements of sets A and B in the union of A and B, the formula is as follows:

(3) $$\mathrm{JI}\left(A,B\right)=\frac{\left|A\cap B\right|}{\left|A\cup B\right|}.$$

In the formula, the larger the JI value, the higher the coincidence degree.

ASD is the average value of the two directional ASD, the formula is as follows:

(4) $$\mathrm{ASD}=\frac{d_{H,\mathrm{avg}}\left(A,B\right)+d_{H,\mathrm{avg}}\left(B,A\right)}{2}.$$

Among them, the directed ASD is the average distance from point A to the nearest neighbor of point B, namely

(5) $$d_{H,\mathrm{avg}}\left(A,B\right)=\frac{1}{\left|A\right|}\sum_{a\in \left|A\right|}\min_{b\in \left|B\right|}d\left(a,b\right).$$

The formula of the HD is as follows:

(6) $$\mathrm{HD}\left(A,B\right)=\max\left(h\left(A,B\right),h\left(B,A\right)\right)$$

(7) $$h\left(A,B\right)=\max\left(\min\left\|a-b\right\|\right).$$

In the formula, the index calculates the surface distance between two equal contours. The smaller the HD value, the closer the distance between A and B, indicating a higher segmentation accuracy. A paired *t*‐test was also performed on DSC of the two models, and *p* < 0.05 is considered a statistically significant difference.

## 3 RESULTS

**FIGURE 2:**
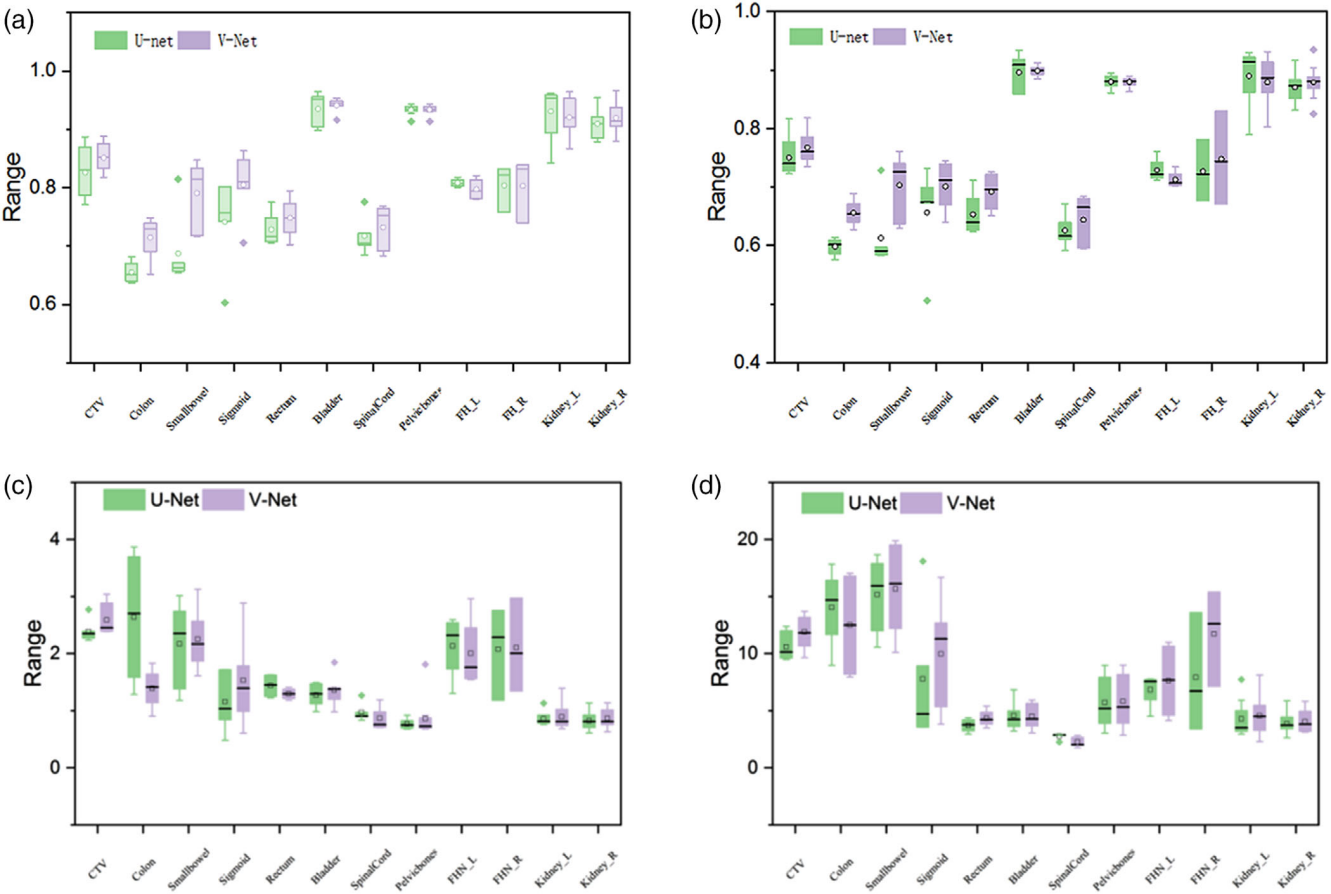
The comparison of the four evaluation indicators of the two networks on delineation of different organs: (a) Dice similarity coefficient (DSC); (b) Jaccard index (JI); (c) average surface distance (ASD); (d) Hausdorff distance (HD). In the figure, the solid dots outside the box represent outliers in this set of data, the solid line inside the box represents the median of this set of data, and the open circles represent the average value of this set of data

The performance indices measured by DSC, JI, ASD, HD on the testing dataset were summarized in Table 1 and Figure 2. The U‐net model results were also included as a comparison to the V‐net model. By comparing the DSC of these two networks, we can clearly see that the DSC of the V‐net model is significantly higher than the U‐net network for the delineation of CTV. In addition, in the delineation of OARs, V‐net performed better for small bowel, colon, sigmoid, etc., and is comparable to U‐net for FHL, FHR, and left kidney.

**TABLE 1:** Mean results of specific delineation of pelvic targets and organs at risk (OARs) for 30 patients in the test dataset. The larger of the two Dice similarity coefficients (DSC) values is shown in bold

	**DSC**			**JI**			**ASD**			**HD**		
**Anatomy**	**U‐net**	**V‐net**	***p*‐Value**	**U‐net**	**V‐net**	***p*‐Value**	**U‐net**	**V‐net**	***p*‐Value**	**U‐net**	**V‐net**	***p*‐Value**
CTV	0.83	**0.85**	*p *= 0.031	0.75	0.77	*p *> 0.05	2.26	2.58	*p *> 0.05	10.08	11.2	*p *> 0.05
Rectum	0.84	**0.85**	*p *> 0.05	0.82	0.83	*p *> 0.05	1.44	1.30	*p *> 0.05	3.72	4.35	*p *> 0.05
Sigmoid	0.72	**0.80**	*p *> 0.05	0.68	0.73	*p *> 0.05	1.16	1.53	*p *> 0.05	7.78	9.96	*p *> 0.05
Small bowel	0.74	**0.79**	*p *= 0.048	0.72	0.73	*p *= 0.019	2.17	2.25	*p *> 0.05	15.16	15.66	*p *> 0.05
Bladder	0.93	**0.94**	*p *> 0.05	0.90	0.90	*p *> 0.05	1.27	1.36	*p *> 0.05	4.58	4.52	*p *> 0.05
Pelvic bones	0.92	0.92	*p *> 0.05	0.87	0.88	*p *> 0.05	0.77	0.86	*p *> 0.05	5.72	5.82	*p *> 0.05
Colon	0.81	**0.82**	*p *= 0.046	0.74	0.76	*p *= 0.008	2.59	1.88	*p *> 0.05	13.69	12.49	*p *> 0.05
Spinal cord	0.72	**0.73**	*p *> 0.05	0.63	0.64	*p *> 0.05	0.98	0.87	*p *> 0.05	2.77	2.26	*p *> 0.05
Femoral Head_L	**0.84**	0.82	*p *> 0.05	0.77	0.74	*p *> 0.05	2.13	2.01	*p *> 0.05	6.85	7.62	*p *> 0.05
Femoral Head_R	**0.82**	0.81	*p *> 0.05	0.76	0.76	*p *> 0.05	2.07	2.11	*p *> 0.05	7.92	11.72	*p *> 0.05
Kidney_L	**0.93**	0.92	*p *> 0.05	0.89	0.88	*p *> 0.05	0.86	0.89	*p *> 0.05	4.28	4.54	*p *> 0.05
Kidney_R	0.91	**0.92**	*p *> 0.05	0.87	0.88	*p *> 0.05	0.83	0.87	*p *> 0.05	3.88	4.05	*p *> 0.05

Abbreviations: ASD, average surface distance; DSC, Dice similarity coefficients; HD, Hausdorff distance (HD); JI, Jaccard index.

**FIGURE 3:**
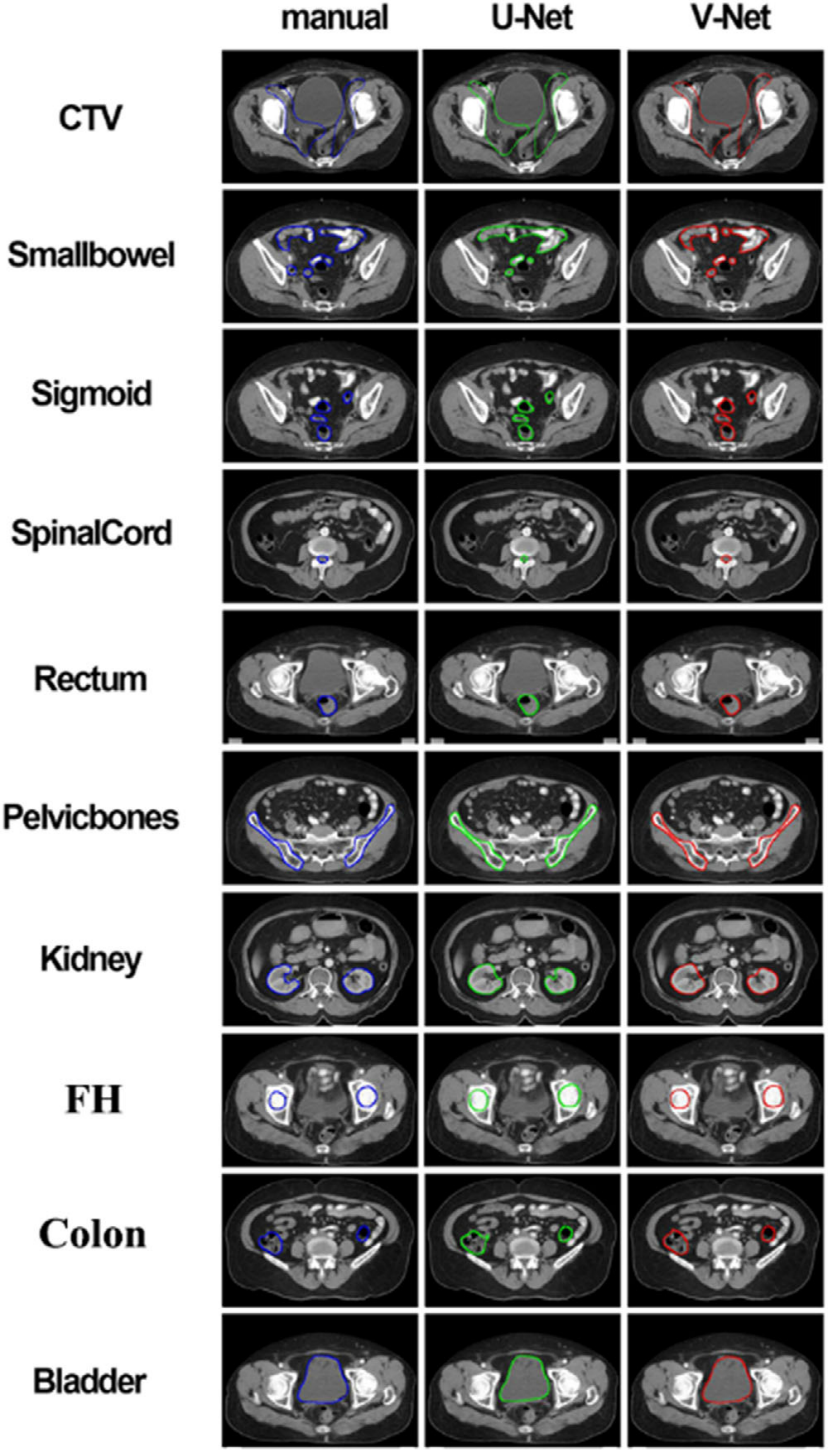
An example case of the delineation results of the pelvic organs by the two networks

The delineation of the pelvic organs by the two networks are shown in Figure 3. The blue line represents the manual contouring by the physician, the green and red lines denote auto‐segmentation by U‐net and V‐net, respectively. As can be seen from Figure 3, the delineation of CTV in both models were close to the ground truth. But for intestinal tissue, the delineation from the V‐net model had more overlapped area with manually delineations, especially that small bowel. Generally, from the comparison in Figure 3 and DSC values in Table 1, the V‐net model achieved better performance than the U‐net model.

The evaluation results of the two models for different dose areas are shown in Table 2. For the specific delineation effect, the comparison of learning results of the two models with the actual dose area in a postoperative radiotherapy plan for a patient is shown in Figure 5. From the evaluation indicators of the learning results of the two models for different dose areas, we can find that for a single‐dose area, the learning results of the two models are not particularly different. But the higher DSC value indicated that the performance of V‐net will be better than the U‐net model. This can also be clearly observed from Figure 5, that the predicted 5 Gy and 15 Gy isodose lines from V‐net had more overlaps with the corresponding isodose lines in the treatment plan. Table 2 also shows that the DSC values of V‐net were significantly better than U‐net for delineation of 5 Gy and 15 Gy isodose lines.

**TABLE 2:** The mean of the specific delineation results for the different dose regions of the 30 patients in the test dataset

	**DSC**			**JI**			**ASD**			**HD**		
**Dose**	**U‐net**	**V‐net**	***p*‐Value**	**U‐net**	**V‐net**	***p*‐Value**	**U‐net**	**V‐net**	***p*‐Value**	**U‐net**	**V‐net**	***p*‐Value**
5 Gy	0.83	**0.88**	*p *= 0.042	0.82	0.87	*p *= 0.037	3.84	3.72	*p *> 0.05	20.14	21.33	*p *> 0.05
10 Gy	0.87	**0.88**	*p *> 0.05	0.83	0.87	*p *> 0.05	5.33	5.22	*p *> 0.05	27.45	34.08	*p *> 0.05
15 Gy	0.78	**0.82**	*p *= 0.039	0.76	0.81	*p *= 0.046	6.25	7.13	*p *> 0.05	36.78	38.86	*p *> 0.05
20 Gy	0.76	**0.78**	*p *> 0.05	0.73	0.77	*p *> 0.05	6.63	7.81	*p *> 0.05	33.29	41.41	*p *> 0.05

Abbreviations: ASD, average surface distance; DSC, Dice similarity coefficients; HD, Hausdorff distance (HD); JI, Jaccard index.

## 4 DISCUSSION

In this study, we proposed to use the V‐net model to auto‐segment cervical cancer target areas and OARs. In order to compare the accuracy of the automatic segmentation of the proposed model, we used four indices including DSC, JI, ASD, and HD to evaluate the performance. During the training process, we also chose different learning rates and loss functions, but the results showed that the learning rate of 0.0003 and the Dice loss function were the best parameters to achieve satisfying performance without being time consuming. According to the results, the auto‐segmentation of other organs including the pelvic bones, bladder, femoral head, and kidney all showed excellent results, except for the slightly poorer DSC of the small bowel and spinal cord. Moreover, the most noteworthy point is that the performance of automatic delineation of cervical cancer CTV by the V‐net model is beyond satisfactory, with a DSC of 0.85, since a DSC larger than 0.7 indicates a high degree of coincidence between the automatic segmentation area and the manual segmentation area, as reported by Andrew et al.^22^

**FIGURE 4:**
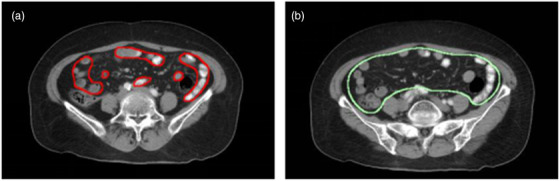
Different delineation standards of the small bowel of the two hospitals. (a) Standard of our hospital, (b) standard of the study referred in Ref^23^

It is not recommended to perform auto‐segmentation in the intestinal area in clinical practice, since the DSCs are between 0.75 and 0.8, as expected. First of all, it is challenging to automatically segment due to the complexity of the pelvic organs, especially the small bowel which can have large differences in size, shape, grayscale, and relative position. Second, compared to studies conducted by Ju et al.^23^ and Kazemifar et al.,^24^ who achieved a DSC around 0.85 delineating the small bowel using other models, the delineation standards of the small bowel of their studies are different from our standard. Figure 4 shows a comparison of our delineation standards of the small bowel to the standard used in the study of Ju et al.^23^ We did not include the soft tissues and other structures covered by the entire small bowel during contouring and only included a single small bowel model, which made our intestinal structure more difficult to be learned by deep learning networks. This results in our intestinal structures’ DSC to be slightly lower than those reported by other literature. Meanwhile, the delineation of the spinal cord did not achieve satisfactory results. Theoretically, due to the contrast or the difference in the HU values between the bone structure and the nearby soft tissues, this structure should be easily distinguished and segmented by the deep learning model. After careful review of the manual segmentation, it was found that the spinal cord was only manually delineated at the target level, which led to incomplete spinal cord structure in model learning.

**FIGURE 5:**
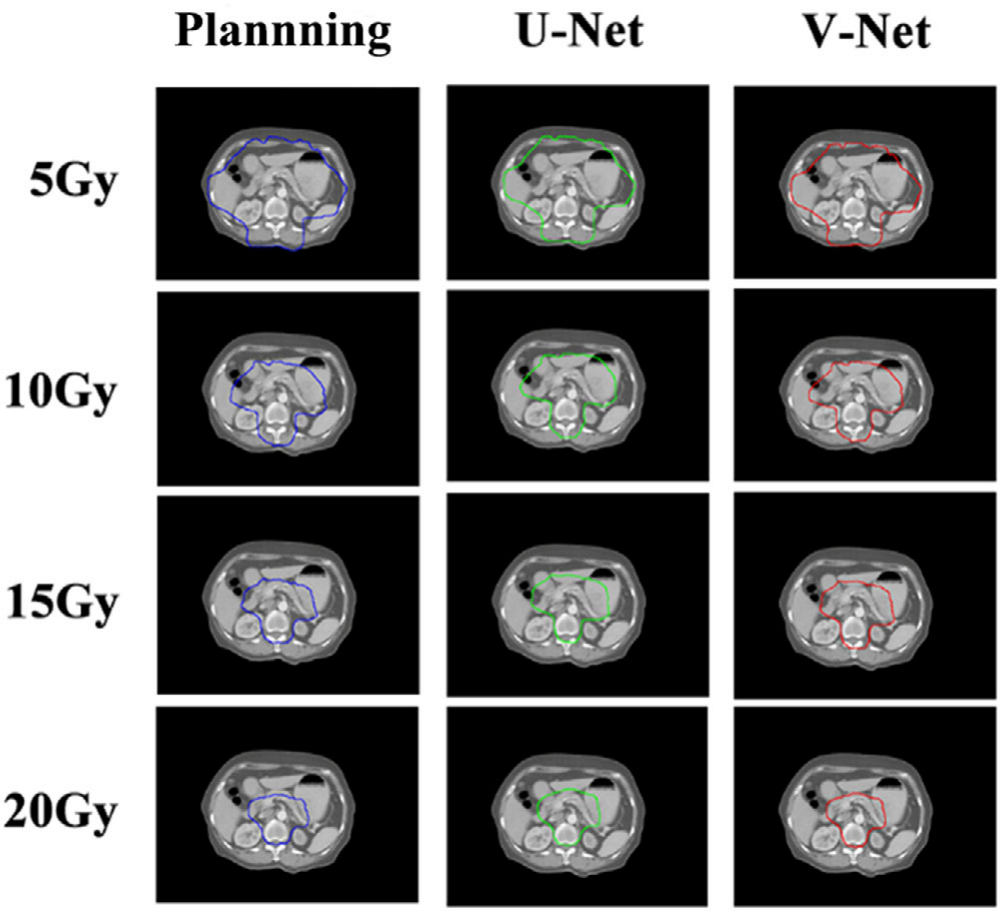
An example of the comparison of predicted isodose lines of U‐net and V‐net to the corresponding isodose lines in the treatment plan

Since published in 2015, because of its excellent processing capabilities for medical images, the U‐net neural network once became the standard for medical image auto‐segmentation. The V‐net model can be regarded as a 3D U‐net. Strictly speaking, the neural network we used belongs to 2.5D rather than 3D CNN. We integrated one upper layer and one lower layer of CT images as a new input data for processing, and formed pseudo‐3D images for learning, reduced the data processing time at the input, thereby enhancing the learning effect and efficient of CT images. From the comparison results, we can see that although V‐net performed slightly better than U‐net in most cases, the delineation performance of U‐net is still comparable with V‐net for the femoral head and kidney. Regarding the pros and cons of the two models, we can analyze from two aspects. From data format point of view, 3D data have one more direction of information than 2D data. Normally, 2D data are expressed as (*x*, *y*) and 3D data are expressed as (*x*, *y*, *z*). Most medical imaging is 3D, which is a superposition of multiple slices. However, due to the different pixel pitches on the *z*‐axis, the 3D data also appear data with different layer thicknesses when layered. From the model point of view, 3D convolution can encode 3D data from three directions (*x*, *y*, *z*), while 2D convolution can only encode 3D data from two directions (*x*, *y*), which is the advantage of 3D convolution. Generally speaking, the parameter of 3D convolution is larger, so the V‐net was only down‐sampled 8 times, compared to 16 times with U‐net. However, due to the matching problem between the amount of data and the amount of model parameters, V‐net may need more data to train to prevent over‐fitting. This is a limitation of our study, since the number of samples we used for model training is only 130. Future work includes more data collection for the model training and testing. The comparison of the performance of the two networks can give guidance on our goal of the 2D and 3D network integration.

DSC is the most commonly used metric in the segmentation field, which measures the volumetric overlap between two segmentation masks. In addition to the DSC, the surface distance metrics, such as HD and ASD, could recognize outliers away from the normal range of OARs.^25^, ^26^ In our study, it is worth noting that the ASD and HD results surface distances are not always consistent with assessments by DSC and JI. This is possibly due to the existence of some false positives outside the normal range of OAR, which only occupy a small number of voxels. These predictions have minimal impact on DSC which measures volume overlap, but they could increase surface distances such as HD and ASD metrics. This inconsistency of DSC and HD is consistent with the results reported by Tang et al.,^25^ who implemented a deep learning framework for OAR delineation in CT images. Their results showed that the DSC values from Ua‐Net were highest for Lens R, SMG L, spinal cord, temporal lobe L/R, but the average 95% HD value of Ua‐net was not the smallest compared to other deep learning models.

Taking into account the need to pay special attention to the dose equivalent of the ovaries in the radiotherapy plan for cervical cancer, we also creatively convert the isodose lines of 5 Gy, 10 Gy, 15 Gy, and 20 Gy in the radiotherapy plan into structures for learning in the neural network.^27^, ^28^ The goal is to predict a suitable location for ovarian transposition, that is, the low‐dose area which approximately could receive less than 5 Gy.^29^ In fact, the isodose line of 5 Gy cannot be referred to the actual 5 Gy. This location after surgery may not receive exactly the predicted dose, due to several factors. First of all, the anatomy of the patient may change after surgery, leading to displacement of isodose lines. Also, as stated in AAPM TG 158 report,^30^ the TPS isodose lines might show large error beyond 3 cm from the field edges, and the TPS tend to underestimate the dose outside the fields. However, it could still give us a good estimate of the low‐dose area. The differences between the prediction of the 5 Gy dose area of 30 patients in the test set using the V‐net network and the corresponding dose areas of the actual radiotherapy plan are barely noticeable. This result has important implications for abdominal radiotherapy in women. According to the study by Yin et al.,^29^ the ovary is an important female reproductive organ, and low‐dose radiation can well protect the female reproductive organs from radiation damage. At the same time, the intestinal organs in the abdomen are also extremely fragile tissues, and the prediction of the low‐dose region will also provide new means for the protection of the abdominal intestinal tract. Our future work will focus on how to apply postoperative dose prediction to provide clinical guidance on ovarian transposition.

## 5 CONCLUSIONS

In this study, we used the V‐net model to automatically delineate cervical cancer CTV and OARs, and compared the delineation results with the U‐net model. In general, the V‐net model based on 3D CNN has better performance on the automatic delineation of the abdomen, although the performance of U‐net on the femoral head and left kidney is comparable with V‐net, there are no significant difference. On the other hand, using a V‐net network can improve the consistency and accuracy of cervical cancer delineation. In addition, we have also used the V‐net network to predict the different dose areas in the radiotherapy plan, which has feasibility of providing clinical advantages in the future.

## CONFLICT OF INTEREST

The authors declare that there is no conflict of interest that could be perceived as prejudicing the impartiality of the research reported.

## AUTHOR CONTRIBUTIONS

Yi Ding and Zhiran Chen contributed equally and are joint first authors. Xiaohong Wang, Ziqi Wang, and Desheng Hu were responsible for the research of the literature; Pingping Ma and Chi Ma compiled the data of the article; Wei Wei, Xiangbin Li, and Xudong Xue provided ideas and ideas; Xiao Wang finally made an overall plan for the article.
